# Transcapillary escape rate of ^125^I-albumin in relation to timing of blood sampling: the need for standardization

**DOI:** 10.1186/s41181-021-00125-0

**Published:** 2021-02-16

**Authors:** Youssef Chahid, Nienke M. G. Rorije, Soufian el Boujoufi, Ron A. A. Mathôt, Liffert Vogt, Hein J. Verberne

**Affiliations:** 1 Department of Radiology and Nuclear Medicine, Amsterdam University Medical Centers, University of Amsterdam, Amsterdam, The Netherlands; 2 Department of Clinical Pharmacy, Amsterdam University Medical Centers, University of Amsterdam, Amsterdam, The Netherlands; 3 Department of Internal Medicine, Section of Nephrology, Amsterdam University Medical Centers, Amsterdam Cardiovascular Sciences, University of Amsterdam, Amsterdam, The Netherlands

**Keywords:** Radioactive iodide labeled human serum albumin, Transcapillary escape rate of albumin, TERalb, Vascular permeability

## Abstract

**Background:**

Increased vascular permeability is an early sign of vascular damage and can be measured with the transcapillary escape rate of albumin (TER_alb_). Although TER_alb_ has a multi-exponential kinetic model, most published TER_alb_ data are based on mono-exponential kinetic models with variation in blood sampling schemes. Aim of this posthoc study was to evaluate the influence of variation in blood sampling schemes and the impact of mono- or bi-exponential analyses on the calculation of TER_alb_. Study participants were part of a cross-over intervention study protocol, investigating effects of sodium loading on blood pressure, endothelial surface layer and microcirculation. Multiple blood samples were drawn between 3 and 60 min after injection of radioactive iodide labeled human serum albumin (rHSA).

**Results:**

In total 27 male participants with 54 measurements were included. For all participants the maximum serum radioactivity was reached within 20 min, while 85% of the participants had their maximum serum activity within 10 min. The TER_alb_ calculated with the subsequently chosen T_20–60 min_ reference scheme (6.19 ± 0.49%/h) was significantly lower compared to the TER_alb_ of the T_3–60 min_, T_5–60 min_, and T_max – 60 min_ schemes. There was no significant difference between the T_20–60 min_ reference scheme and the T_10–60 min_ and T_15–60 min_ schemes. Bi-exponential kinetic modeling did not result in significant different observations compared to the mono-exponential kinetic analysis.

**Conclusions:**

As there is variation in the timing of the maximum serum radioactivity of rHSA, blood sampling schemes starting before 10 min after administration of rHSA will result in a significant overestimation of TER_alb_. In addition, variation in kinetic modeling did not result in significant changes in TER_alb_. Therefore, we emphasize the need to standardize TER_alb_ and for practical and logistical reasons advocate the use of a mono-exponential model with blood sampling starting 20 min after rHSA administration.

## Background

Diabetes mellitus and hypertension are characterized by an increased risk of vascular complications. An early sign of vascular damage is increased vascular permeability, which can be determined by the transcapillary escape rate of albumin (TER_alb_) (Broekhuizen et al., 2010).

TER_alb_ is the rate in which intravenous albumin escapes from the intravascular to the extravascular volume in the first hour after injection of radioactive iodide labeled human serum albumin (rHSA) (Parving, 1975). The pharmacokinetics of rHSA could be described as the sum of three exponential components, with respective half-life’s of 6.8 h, 1.29 days and 19.4 days (Berson et al., 1953; Bauman et al., 1955; CIS bio International, 2010). The disappearance of rHSA, in the first hour after injection, could be described as a bi-exponential decay curve with a inflection point after approximately 10 min (Margarson & Soni, 2002).

Despite the fact that rHSA has a multi-compartment kinetic model, all published TER_alb_ data analyses are based on a mono-exponential kinetic model. This mono-exponential TER_alb_ model has four assumptions: the rHSA behaves like endogenous albumin; the albumin metabolism is in steady state during the TER_alb_ test; rHSA has a mono-exponential blood pool elimination during the first hour after injection, with a rate constant equal to that of time zero; the initial blood pool elimination reflects extravasation and is not influenced by the rHSA metabolism rate (Parving & Gyntelberg, 1973).

The original protocol of Parving et al. describes that a small amount of I-125 or I-131 labeled rHSA is injected in an arm vein, and eight venous blood samples were drawn from the contralateral arm at 10, 15, 20, 30, 40, 50, 55, and 60 min after the injection. The radioactivity of the rHSA in each blood sample was measured in duplicate. The TER_alb_ was calculated and expressed as the percentage decline of radioactivity during the first hour (%/h) (Parving & Gyntelberg, 1973).

However most studies using TER_alb_ values show variation in sampling schemes ranging from 3 to 13 blood samples (Margarson & Soni, 2002; Jensen et al., 1992). Some of the schemes started already 1 min after the injection of rHSA, while others started blood sampling 20 min after the administration of rHSA (Margarson & Soni, 2002; Norberg et al., 2015).

This variation in sampling schemes does impact the calculated TER_alb_. Sampling schemes that started 5 min after administration found TER_alb_ in the range of 6.9–9.1%/h (Dell'omo et al., 2006; Dell'Omo et al., 2000; Haskell et al., 1997; Pedrinelli et al., 2000; Pedrinelli et al., 1999; van Eijk et al., 2005; Rorije et al., 2018). While studies which started sampling 10 min after injection found a lower TER_alb_ of approximately 5.5%/h (Parving, 1975; Jensen et al., 1992; Nannipieri et al., 1997; Jensen, 1995; Nannipieri et al., 1995; Staberg et al., 1982; Zietse et al., 1995). These differences in TER_alb_ were not related to differences in patient population, but are in line with the multi-exponential kinetics of rHSA.

As the use of TER_alb_ for clinical research seems to gain in popularity, standardization of the technique is essential: i.e. reducing variation in performing the test and thereby reducing variation in the test result. As we observed large variations between different publications in TER_alb_ sampling schemes and most likely thereby variation in TER_alb_ results, we therefore aimed to study the influence of different sampling schemes and the use of a mono- or bi-exponential analysis on the calculation of TER_alb_.

## Methods

### Study population and study design

Selected participants of this post hoc study were part of a cross-over intervention study protocol investigating whether an acute intravenous sodium load, as compared to a chronic dietary sodium load, differs in its effects on blood pressure, the endothelial surface layer and microcirculation (Rorije et al., 2018). Participants included healthy men, and both male type 1 diabetes mellitus and hereditary multiple exostosis patients (i.e., patients with, respectively, acquired and genetically determined glycocalyx changes) (Mooij et al., 2014). Exclusion criteria were hypertension (≥ 140/90 mmHg), obesity (body mass index (BMI) ≥ 30 kg/m^2^), history of primary hyperlipoproteinemia, coagulation disorders, and renal or cardiovascular diseases. All participants were randomized to a low sodium diet (LSD, < 50 mmol Na^+^ daily) or to a high sodium diet (HSD, > 200 mmol Na^+^ daily) for 8 days, separated by a crossover period of at least 1 week. The study was performed at the Amsterdam UMC, location AMC, Amsterdam, The Netherlands. All participants provided written informed consent and approval was obtained from the local ethics committee. The trial is registered in the Netherlands Trial Register (NTR4095 and NTR4788).

### Transcapillary escape rate of rHSA

An intravenous (IV) bolus of saline solution with rHSA labeled with 100 kBq I-125 was administered in a cubital vein. Blood samples were drawn from the contralateral arm at baseline and between 3 and 60 min after injection of rHSA. Radioactivity in plasma was measured in duplicate with a Wizard2 2480 automatic gamma counter (PerkinElmer, Waltham, Massachusetts, USA) with a coefficient of variation of < 3%. The routine quality controls of the gamma counter were performed according to the standard GLP features of PerkinElmer, including detector energy resolution, background, absolute - and relative detector efficiency, detector stability probability and calibration.

The TER_alb_ was calculated with PKSolver, a free Microsoft Excel add-in for pharmacokinetic (PK) and pharmacodynamic (PD) data analysis (Zhang et al., 2010). PKSolver has been validated and has been used in different PK/PD studies (Zhang et al., 2010; Kulo et al., 2017; de Velde et al., 2016; Nezic et al., 2014; Balakumar et al., 2013; Wenstedt et al., 2020).

TER_alb_ was expressed as percentage decline in plasma radioactivity per hour (%/h). The TER_alb_ calculation with PKSolver was performed for an IV bolus administration. The formula used for the calculation of TER_alb_ was:

$$ {\mathrm{TER}}_{\mathrm{alb}}=\left({\mathrm{A}}_{0\ \min }-{\mathrm{A}}_{60\ \min}\right)/{\mathrm{A}}_{0\ \min } $$

The predicted activity of rHSA at T_0 min_ (A _0 min_) and at T_60 min_ (A _60 min_) were calculated by PKSolver (Microsoft Excel 2016) based on a mono- and bi-exponential kinetic model. This program also calculated the correlation coefficient (R) between the observed and predicted data.

### Sampling schemes

After acquiring the PK curves of rHSA, we calculated the TER_alb_ according the following simulated blood sampling schemes:

- T_3–60 min_: 3, 4, 5, 10, 15, 20, 30, 45, and 60 min
- T_5–60 min_: 5, 10, 15, 20, 30, 45, and 60 min
- T_10–60 in_: 10, 15, 20, 30, 45, and 60 min
- T_15–60 min_: 15, 20, 30, 45, and 60 min
- T_20–60 min_: 20, 30, 45, and 60 min
- T_max – 60 min_: from individual A _max_ till 60 min

All blood samples before A _max_ of the PK curves were excluded for the calculation of TER_alb,_ irrespective of the sampling scheme.

### Statistics

The effect of different blood sampling schemes on the TER_alb_ values were analyzed by fitting a mixed model as implemented in IBM SPSS Statistics (version 26, IBM, USA). This mixed model uses a compound symmetry covariance matrix and is fitted using maximum likelihood. In the absence of missing values, this method results in the same *p* values as multiple comparisons tests (e.g. repeated measures ANOVA) that are less able to deal with missing values. Therefore, in the presence of missing values, the results can be interpreted like repeated measures ANOVA (Harrison et al., 2018). We used Bonferroni correction as post hoc test and *p* values < 0.05 were considered statistically significant. Results were reported as mean ± standard error of the mean (SEM). Bland-Altman plots were used to evaluate the level of agreement between two different blood sample schemes.

## Results

### Patient demographics

In total 27 men were included resulting in 54 PK curves (27 linked to the LSD and 27 linked to the HSD), based on 486 (54*9 samples) blood sample analyses. The study population consisted of 12 healthy volunteers, 8 diabetes mellitus type I patients, and 7 patients with hereditary multiple exostoses. All volunteers were between 18 and 38 years old with a median age of 24 (range 18–38 years). Other characteristics of study participants are displayed in Table 1.

**Table 1:** Characteristics of study participants

Characteristics of participants	Result
Health status (n)	27
Healthy	12
DM type I	8
HME^a^	7
Age (years median, range)	24 (18–38)
Healthy	21 (18–31)
DM type I	26 (21–37)
HME	21 (19–38)
Length (cm ± SD^b^)	183.7 (5.8)
Healthy	185.6 (6.3)
DM type I	184.3 (5.0)
HME	179.9 (4.1)
Weight (kg ± SD)	77.0 (7.6)
Healthy	75.7 (6.8)
DM type I	77.4 (9.4)
HME	78.8 (7.3)
BMI (kg/m^2^ ± SD)	22.9 (2.5)
Healthy	22.0 (2.2)
DM type I	22.8 (2.5)
HME	24.4 (2.9)
eGFR^c^ (ml/min ± SD)	118.1 (10.3)
Healthy	114.7 (12.1)
DM type I	120.3 (9.6)
HME	121.6 (6.3)

^a^HME (hereditary multiple exostoses), ^b^SD (standard deviation), ^c^eGFR based on CKD-EPI equation

**Fig. 1:**
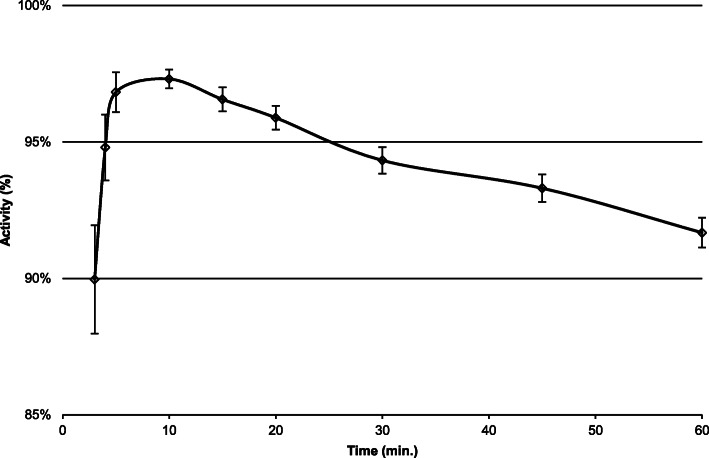
Relative plasma activity in the first hour after administration of rHSA (Tmax is 100% activity for each individual participant)

The blood serum disappearance of rHSA of the study participants is shown in Fig. 1. The pharmacokinetic graphic shows a bi-exponential slope of decay curve with inflexion point at 15–20 min.

### T_max_ after rHSA administration

**Fig. 2:**
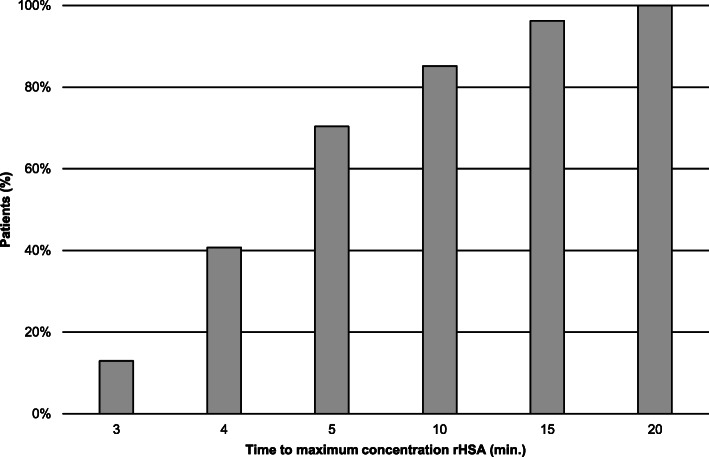
Percentage of participants reaching serum maximum rHSA activity after administration of rHSA (T_max_)

The T_max_ after rHSA administration showed a large inter-individual variability (Fig. 2). The mean T_max_ was 6.9 ± 0.6 min. In 85% of the participants A_max_ of rHSA was reached within 10 min, while T_max_ was reached at 20 min after administration for all participants without an effect of subject category (HME, DM type 1 or healthy volunteer) or the diet followed (LSD vs HSD). Therefore T_20–60 min_ was used as the reference scheme. The mean TER_alb_ values of the other time schemes were compared to the reference scheme T_20–60 min_ based on mono-exponential kinetic analysis.

### TER_alb_ based on mono-exponential kinetic analysis

**Fig. 3:**
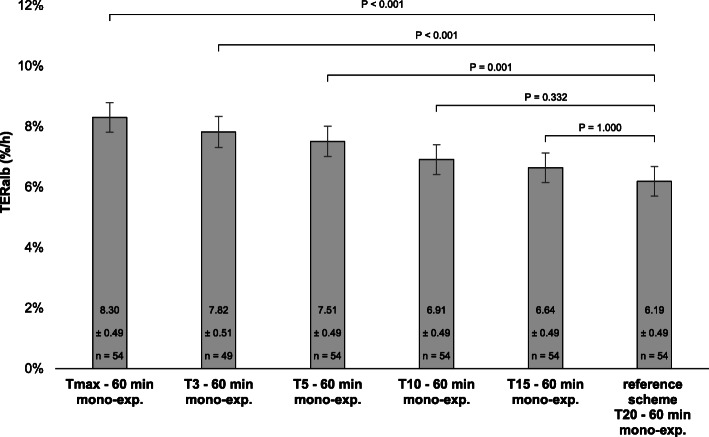
TER_alb_ for the different blood sampling time schemes based on a mono-exponential kinetic analysis and compared with the T_20–60 min_ reference scheme

The reference T_20–60 min_ scheme included 54 of the 54 PK curves. The mean TER_alb_ of the T_max – 60 min_ scheme resulted in the highest calculated TER_alb_: 8.30 ± 0.49%/h (Fig. 3). The TER_alb_ calculated with the T_20–60 min_ reference scheme (6.19 ± 0.49%/h) was significantly lower compared to the TER_alb_ of the T_3–60 min_ (mean difference = − 1.63%/h, CI = − 2.65 – − 0.61%/h, *p* < 0.001), T_5–60 min_ (mean difference = − 1.32%/h, CI = − 2.27 – − 0.36%/h, *p* = 0.001), and T_max – 60 min_ (mean difference = − 2.11%/h, CI = − 3.04 – − 0.48%/h, p < 0.001) schemes. There were no significant difference between the mean TER_alb_ of the T_20–60 min_ reference scheme and the T_10–60 min_ and T_15–60 min_ scheme.

### TER_alb_ based on bi-exponential kinetic analysis

**Fig. 4:**
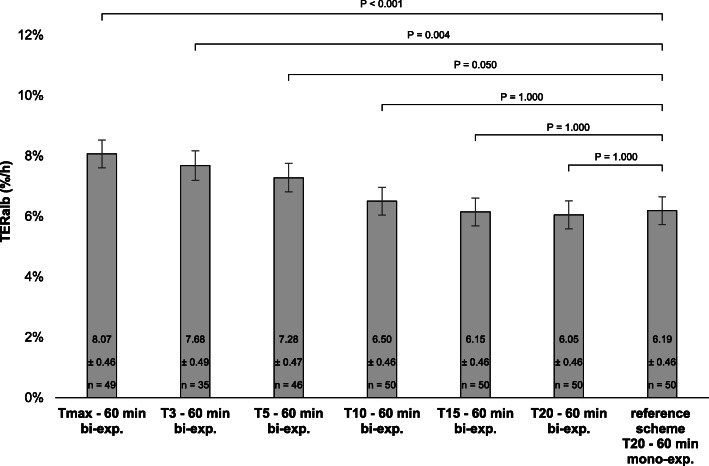
TER_alb_ for the different blood sampling schemes based on a bi-exponential kinetic model, compared with the T_20–60 min_ reference scheme

Using a bi-exponential analysis according to the T_20–60 min_ scheme did not result in significant different TER_alb_ values when compared to the mono-exponential analysis based T_20–60 min_ reference scheme (respectively 6.19 ± 0.46%/h vs. 6.05 ± 0.46%/h, *p* = 1.000). The mean TER_alb_ of the reference T_20–60 min_ scheme was significantly lower compared to the mean TER_alb_ of the bi-exponential kinetic analysis of the T_3–60 min_ (mean difference = − 1.49%/h, CI = − 2.68 – − 0.29%/h, *p* = 0.004), T_5–60 min_ (mean difference = − 1.09%/h, CI = − 2.18 – − 0.01%/h, *p* = 0.050), and T_max – 60 min_ (mean difference = − 1.87%/h, CI = − 2.95 – − 0.80%/h, *p* < 0.001) schemes. There were no significant difference between the mean TER_alb_ of the T_20–60 min_ reference scheme and the T_10–60 min_ and T_15–60 min_ schemes based on bi-exponential kinetic analysis (Fig. 4).

**Fig. 5:**
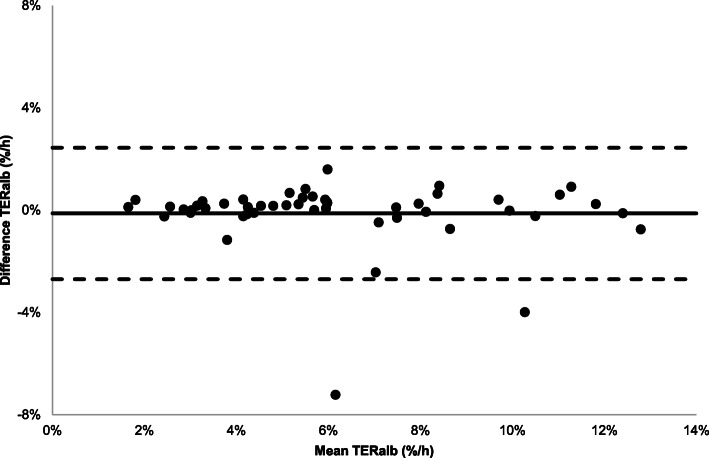
Bland-Altman plot with differences between TER_alb_ values calculated according T_20–60 min, bi-exp._ scheme and the T_20–60 min_ reference scheme. Solid line: bias (− 0.1%/h) between the two sampling schemes, dotted lines: 95% limits of agreement (− 2.7–2.4%/h)

Figure 5 shows the Bland-Altman plot with agreement between the bi-exponential analysis based on T_20–60 min_ scheme and T_20–60 min_ reference scheme. The TER_alb_ showed a bias of − 0.1%/h between the different time schemes without a significant trend over the data range (1.7–12.8%/h) and with a consistent variability over the data range.

## Discussion

To our knowledge, this study is the first to examine the influence of different blood sampling schemes and the impact of mono- or bi-exponential analyses on the calculation of TER_alb_. Our findings emphasize the necessity to standardize TER_alb_ calculations.

We found that the TER_alb_ became lower when blood sample collection started later. This phenomenon has been reported previously (Margarson & Soni, 2002). In this context it is remarkable that the majority of published studies used a fixed time sampling scheme with the first blood sampling within 10 min (Dell'omo et al., 2006; Dell'Omo et al., 2000; Haskell et al., 1997; Pedrinelli et al., 2000; Pedrinelli et al., 1999; van Eijk et al., 2005; Rorije et al., 2018). This practice will have caused a overestimation of the reported TER_alb_. In addition, this makes the reported findings based on TER_alb_ difficult to reproduce and troublesome to extrapolate. Especially when TER_alb_ values of different sampling schemes are compared with each other.

Although the blood serum disappearance of rHSA should be described as a bi-exponential kinetic model, as shown in Fig. 1, the mean TERalb values between mono- and bi-exponential analysis were not significant different. Therefore, we concluded that the mono-exponential kinetic analysis, which is common used for TER_alb_ analysis, is a robust and easy to use approach to calculate the TER_alb_ in the daily practice.

Our data showed that biodistribution of rHSA seems to be complete after 15–20 min. Apparently rHSA may need up to 20 min to reach an equilibrium. This inter-individual variation may be explained by the rate of lymphatic return or redistribution into the hepatic and splenic interstitium (Margarson & Soni, 2002; Henriksen & Schlichting, 1981). To minimize the number of blood samples, we advocate the use a mono-exponential model with blood sampling starting 20 min after rHSA administration for the daily practice. For scientific purposes, we suggest to use the T_max_ scheme to correct for the inter- and intra-individual variability. It should be noted that these TERalb values are significant higher compared to the daily practice scheme.

This study has several limitations that need to be addressed. First, we pooled data, because no differences between healthy participants, type 1 diabetes mellitus and hereditary multiple exostosis patients were detected. One would have expected higher TERalb values in type 1 diabetes mellitus patients. However, our study included young male with uncomplicated type 1 diabetes without albuminuria. Likely, at this stage of the disease, TERalb is still unaffected. Secondly, we did not collect any blood samples after T_60 min_. Blood sampling for longer time periods after administration, for example up to 24 h after rHSA injection, could have helped in better understanding the kinetics of rHSA blood clearance.

## Conclusions

To our knowledge, this study examined for the first time whether different blood sampling schemes impact TER_alb_ values. We found significant differences between the blood sampling schemes which will cause bias in reporting TER_alb_ and makes it difficult to reproduce and extrapolate outcomes of TER_alb_.

As there is a large variation in the timing of the maximum serum radioactivity of rHSA, blood sampling schemes starting before 10 min after administration of rHSA will result in a significant overestimation of TER_alb_. In addition, variation in mono- or bi-exponential kinetic modeling did not result in significant changes in TER_alb_. Therefore, we emphasize the need to standardize TER_alb_ and for practical and logistical reasons advocate the use of a mono-exponential model with blood sampling starting 20 min after rHSA administration.

## Data Availability

The datasets used and/or analysed during the current study are available from the corresponding author on reasonable request.

## Abbreviations

rHSA: radioactive iodide labeled human serum albumin
TERalb: Transcapillary escape rate of albumin
Tmax: Time to peak drug concentration
Cmax: Peak drug concentration
I-125: Iodine-125
I-131: Iodine-131
BMI: Body mass index
LSD: Low sodium diet
HSD: High sodium diet
GLP: Good Laboratory Practice

## References

[CR1] Balakumar K, Raghavan CV, Selvan NT, Prasad RH, Abdu S (2013). Self nanoemulsifying drug delivery system (SNEDDS) of rosuvastatin calcium: design, formulation, bioavailability and pharmacokinetic evaluation. Colloids Surf B Biointerfaces.

[CR2] Bauman A, Rothschild MA, Yalow RS, Berson SA (1955). Distribution and metabolism of I131 labeled human serum albumin in congestive heart failure with and without proteinuria. J Clin Invest.

[CR3] Berson SA, Yalow RS, Schreiber SS, Post J (1953). Tracer experiments with I131 labeled human serum albumin: distribution and degradation studies. J Clin Invest.

[CR4] Broekhuizen LN, Lemkes BA, Mooij HL, Meuwese MC, Verberne H, Holleman F (2010). Effect of sulodexide on endothelial glycocalyx and vascular permeability in patients with type 2 diabetes mellitus. Diabetologia.

[CR5] CIS bio international. Summary of product characteristics of SERALB-125. France: CIS bio international; 2010.

[CR6] de Velde F, de Winter BC, Koch BC, van Gelder T, Mouton JW (2016). Consortium C-N. non-linear absorption pharmacokinetics of amoxicillin: consequences for dosing regimens and clinical breakpoints. J Antimicrob Chemother.

[CR7] Dell’Omo G, Bandinelli S, Penno G, Pedrinelli R, Mariani M (2000). Simvastatin, capillary permeability, and acetylcholine-mediated vasomotion in atherosclerotic, hypercholesterolemic men. Clin Pharmacol Ther.

[CR8] Dell'omo G, Penno G, Pucci L, Lucchesi D, Fotino C, Del Prato S (2006). ACE gene insertion/deletion polymorphism modulates capillary permeability in hypertension. Clin Sci.

[CR9] Harrison XA, Donaldson L, Correa-Cano ME, Evans J, Fisher DN, Goodwin CED (2018). A brief introduction to mixed effects modelling and multi-model inference in ecology. Peer J.

[CR10] Haskell A, Nadel ER, Stachenfeld NS, Nagashima K, Mack GW (1997). Transcapillary escape rate of albumin in humans during exercise-induced hypervolemia. J Appl Physiol.

[CR11] Henriksen JH, Schlichting P (1981). Increased extravasation and lymphatic return rate of albumin during diuretic treatment of ascites in patients with liver cirrhosis. Scand J Clin Lab Invest.

[CR12] Jensen EW, Bryde Andersen H, Nielsen SL, Christensen NJ (1992). Long-term smoking increases transcapillary escape rate of albumin. Scand J Clin Lab Invest.

[CR13] Jensen JS (1995). Renal and systemic transvascular albumin leakage in severe atherosclerosis. Arterioscler Thromb Vasc Biol.

[CR14] Kulo A, Smits A, Maleskic S, Van de Velde M, Van Calsteren K, De Hoon J (2017). Enantiomer-specific ketorolac pharmacokinetics in young women, including pregnancy and postpartum period. Bosn J Basic Med Sci.

[CR15] Margarson MP, Soni NC (2002). Effects of albumin supplementation on microvascular permeability in septic patients. J Appl Physiol.

[CR16] Mooij HL, Cabrales P, Bernelot Moens SJ, Xu D, Udayappan SD, Tsai AG (2014). Loss of function in heparan sulfate elongation genes EXT1 and EXT 2 results in improved nitric oxide bioavailability and endothelial function. J Am Heart Assoc.

[CR17] Nannipieri M, Penno G, Rizzo L, Pucci L, Bandinelli S, Mattei P (1997). Transcapillary escape rate of albumin in type II diabetic patients. The relationship with microalbuminuria and hypertension. Diabetes Care.

[CR18] Nannipieri M, Rizzo L, Rapuano A, Pilo A, Penno G, Navalesi R (1995). Increased transcapillary escape rate of albumin in microalbuminuric type II diabetic patients. Diabetes Care.

[CR20] Nezic L, Derungs A, Bruggisser M, Tschudin-Sutter S, Krahenbuhl S, Haschke M (2014). Therapeutic drug monitoring of once daily aminoglycoside dosing: comparison of two methods and investigation of the optimal blood sampling strategy. Eur J Clin Pharmacol.

[CR21] Norberg A, Rooyackers O, Segersvard R, Wernerman J (2015). Albumin kinetics in patients undergoing major abdominal surgery. PLoS One.

[CR22] Parving HH (1975). Microvascular permeability to plasma proteins in hypertension and diabetes mellitus in man--on the pathogenesis of hypertensive and diabetic microangiopathy. Dan Med Bull.

[CR23] Parving HP, Gyntelberg F (1973). Transcapillary escape rate of albumin and plasma volume in essential hypertension. Circ Res.

[CR24] Pedrinelli R, Dell'Omo G, Bandinelli S, Penno G, Mariani M (2000). Transvascular albumin leakage and forearm vasodilatation to acetylcholine in essential hypertension. Am J Hypertens.

[CR25] Pedrinelli R, Penno G, Dell’Omo G, Bandinelli S, Giorgi D, Di Bello V (1999). Microalbuminuria and transcapillary albumin leakage in essential hypertension. Hypertension.

[CR27] Rorije NMG, Olde Engberink RHG, Chahid Y, van Vlies N, van Straalen JP, van den Born BH (2018). Microvascular permeability after an acute and chronic salt load in healthy subjects: a randomized open-label crossover intervention study. Anesthesiology.

[CR28] Staberg B, Worm AM, Rossing N, Brodthagen H (1982). Microvascular leakage of plasma proteins after PUVA and UVA. J Invest Dermatol.

[CR29] van Eijk LT, Pickkers P, Smits P, van den Broek W, Bouw MP, van der Hoeven JG (2005). Microvascular permeability during experimental human endotoxemia: an open intervention study. Crit Care.

[CR30] Wenstedt EFE, Rorije NMG, Olde Engberink RHG, van der Molen KM, Chahid Y, Danser AHJ (2020). Effect of high-salt diet on blood pressure and body fluid composition in patients with type 1 diabetes: randomized controlled intervention trial. BMJ Open Diabet Res Care.

[CR31] Zhang Y, Huo M, Zhou J, Xie S (2010). PKSolver: an add-in program for pharmacokinetic and pharmacodynamic data analysis in Microsoft excel. Comput Methods Prog Biomed.

[CR32] Zietse R, Derkx FH, Weimar W, Schalekamp MA (1995). Effect of atrial natriuretic peptide on renal and vascular permeability in diabetes mellitus. J Am Soc Nephrol.

